# Weekly use of fluconazole as prophylaxis in haematological patients at risk for invasive candidiasis

**DOI:** 10.1186/s12879-014-0573-5

**Published:** 2014-11-11

**Authors:** Danielle Vuichard, Maja Weisser, Christina Orasch, Reno Frei, Dominik Heim, Jakob R Passweg, Andreas F Widmer

**Affiliations:** Division of Infectious Diseases and Hospital Epidemiology, University Hospital Basel, Petersgraben 4, Basel, 4031 Switzerland; Department of Clinical Epidemiology and Biostatistics, McMaster University, Hamilton, ON Canada; Infectious Diseases and Internal Medicine Practice, Klinik St. Anna, St. Anna-Strasse 32, Lucerne, 6006 Switzerland; Division of Clinical Microbiology, University Hospital Basel, Petersgraben 4, Basel, 4031 Switzerland; Division of Haematology, University Hospital Basel, Petersgraben 4, Basel, 4031 Switzerland

## Abstract

**Background:**

The goal was to determine whether one medical centres’ unique antifungal prophylactic regimen for patients at high risk for invasive candidiasis because of their haematological malignancies, haematopoietic stem cell transplants, or high-dose chemotherapy might lead ultimately to a higher incidence of infection, to increasing fluconazole resistance, or to a shift in the predominant strain of *Candida* in invasive fungal episodes.

**Methods:**

Data were collected retrospectively, for a ten-year period from ONKO-KISS surveillance records, and from hospital, medical, and pharmacy records and then evaluated with respect to incidence of fungal infection episodes, emergence of antifungal drug resistance, and predominance of specific *Candida* strains in isolate cultures. Fisher’s exact test and linear regression were used to compare minimum inhibitory concentrations and to compare the incidence of different *Candida* isolates, respectively.

**Results:**

The incidence of infection remained quite stable over 10 years with a median of 0.67 episodes/1000 bed days. Overall, *Candida glabrata* was the predominant species with 29% followed by *C. albicans* and *C. krusei* (14% each). No significant increment of non-albicans *Candida* species with decreased fluconazole susceptibility was perceived over this decade.

**Conclusions:**

Once weekly administration of 400 mg of fluconazole to prevent candidaemia appears to have no negative impact on the efficacy as a prophylaxis when compared to standard of care (400 mg of fluconazole daily).

**Electronic supplementary material:**

The online version of this article (doi:10.1186/s12879-014-0573-5) contains supplementary material, which is available to authorized users.

## Background

The incidence of invasive candidiasis in patients who have haematological malignancies or who are recipients of haematopoietic stem cell transplants (HSCTs) has decreased over the past few decades, in particular because of the antifungal prophylactic use of triazoles. However, invasive candidiasis is the most common invasive fungal infection in the early phase of recovery after HSCT, and remains the second most common invasive fungal infection after invasive aspergillosis in patients with graft-versus-host disease (GvHD). Invasive candidiasis still causes significant morbidity and attributable mortality in these patients; therefore, adequate antifungal prophylaxis is crucial for them [1]-[3]. Finding an optimum antifungal prophylaxis remains an on-going goal. Studies have suggested that fluconazole doses below 400 mg/day may reduce only superficial fungal infections but have limited efficacy in preventing invasive candidiasis [4],[5]. However, a small retrospective analysis in 2001 of antifungal prophylaxis using 100–200 mg of fluconazole per day in patients undergoing bone marrow transplantation showed the incidence of *C. glabrata* and *C. krusei* breakthrough infections to be similar to the incidence of breakthrough infections reported with doses of 400 mg of fluconazole per day and a randomized controlled trial conducted with children and adult patients undergoing HSCT comparing 200 mg versus 400 mg of fluconazole per day demonstrated similar efficacy in reducing superficial and systemic infection with *Candida* species with both regimens [6],[7].

Once daily dosing of 400 mg of fluconazole has been used as prophylaxis against invasive candidiasis for patients in profound neutropenia since many years and is a result of various randomized placebo-controlled trials [8]-[10]. The Centers for Disease Control and Prevention, Atlanta, USA (CDC) guidelines advise against the use of lower fluconazole dosages (between 100–200 mg/day) as antifungal prophylaxis for HSCT recipients [11].

On the reverse isolation haematology unit (RIHU) at this hospital the experience with daily non-absorbable nystatine and amphotericine B was not satisfying with patient adherence reported to be low. Data with haematological patients in neutropenia have shown that 50 mg of fluconazole daily effectively reduced colonizing and superficial candida infections [12],[13]. Based on these results, and data derived from HIV patients, which showed that weekly dosages of 150 mg sufficiently prevent oropharyngeal candidiasis [14],[15], a once weekly prophylaxis of fluconazole 400 mg was thought to equally reduce colonizing *Candida* species in our haematology patients and therefore, prevent invasive infection from this species during severe immunosuppression with the advantage of better tolerance and acceptance by the patient, less drug interactions, and a minor shift towards invasive non-albicans *Candida* species.

For this reason, the division of haematology instituted a change in the antifungal prophylaxis regimen policy in the 1990s for patients during aplasia after they had undergone high-intensity chemotherapy, or an autologous HSCT, and for patients who were immunosuppressed after undergoing an allogeneic HSCT. The policy specified administering 400 mg of fluconazole once a week, rather than once per day. This extraordinary prophylaxis was then combined with a diagnostic driven pre-emptive treatment strategy in cases of suspected fungal infection, at which time fluconazole prophylaxis is replaced by antifungal treatment with caspofungin or voriconazole. Regarding the incidence of invasive fungal infections overall, this latter strategy thus far has proven safe for patients undergoing allogeneic HSCT at the medical centre [16].

There are, however, unanswered questions with respect to the possible emergence of resistance to fluconazole over time and the persistent lack of evidence of effectiveness for this exceptional prophylactic regimen. In contrary, evidence has been increasing that routine prophylaxis with 400 mg fluconazole per day may lead either to an increase in fluconazole resistance, and an increase in the minimum inhibitory concentrations (MICs) of antifungal agents that are necessary to be effective against *Candida* species or to a shift from predominantly *Candida albicans* toward other less fluconazole susceptible *Candida* species in episodes of invasive candidiasis [17],[18]. The elimination half-life of fluconazole is around 30 hours. This results in sub-inhibitory concentrations after only a few days, implicating that there might be an even greater possibility of increasing emergence of resistance at fluconazole doses of 400 mg per week.

Therefore, the goal of this study was to determine whether the hospital’s antifungal prophylactic regimen for patients at high risk for invasive candidiasis might be leading to increased resistance to fluconazole or to a shift toward other strains of *Candida* sp. and, thus, to gradually reduce efficacy for these patients.

## Methods

The setting for this study is one of the three largest stem cell transplantation units in Switzerland at a 700-bed primary and tertiary care hospital performing both allogeneic and autologous HSCT. The RIHU at the University Hospital Basel, Switzerland is located in a closed wing and provides a protected environment for patients that are undergoing HSCTs or high-dose chemotherapy. The unit is equipped with laminar air flow, has restricted access, and has a dedicated staff that wears gowns, masks, and gloves whenever entering the patients’ rooms. Patients admitted to the RIHU are placed in single-occupancy rooms until neutrophil recovery.

We analysed a cohort of patients hospitalized on RIHU between 1 January 2003 and 31 December 2012. The study used data collected within the framework of the medical centre’s participation in the German nosocomial infection surveillance system (Krankenhaus Infektions Surveillance System), which was initiated in 2000 and focuses on nosocomial sepsis and pneumonia in patients with haematological malignancies (known as ONKO-KISS) and prospectively collects data from patients during their episode of neutropenia [19]. The RIHU of the University Hospital Basel has participated in ONKO-KISS since July 2003. The characteristics of the remaining patients hospitalised in RIHU from January 2003 through June 2003 are therefore missing. Case report forms provided the prospectively recorded ONKO-KISS information including demographics, underlying disease and treatment regimens. Detailed information about the patients with invasive candidiasis was retrieved from the hospital’s medical records and from the hospital’s microbiology laboratory as not all these patients were concomitantly in neutropenia and/or underwent HSCT during their hospitalisation with invasive candidiasis and were, therefore, not recorded in ONKO-KISS. Data included general demographic information, the duration of RIHU stays, information about Human Leukocyte Antigen (HLA)-typing and donor relatedness, the number and type of HSCT, underlying disease or condition, treatment regimens, serostatus of cytomegalovirus (CMV) and CMV infection, incidence of graft-versus-host disease (GvHD), incidence of invasive candidiasis, duration of neutropenia, duration of hospitalisation in the Intensive Care Unit (ICU), and deaths within 30 days after a diagnosis of invasive candidiasis as well as results of cultures that identified invasive candidiasis strains, the date of specimen entry into the laboratory, and the corresponding resistance testing.

Data on the use of antifungal consumption were available from the hospital’s central pharmacy from January 2004 onward. Antifungal consumption was measured as a defined daily dose (DDD) per 100 patient days (PD). Defined daily doses (DDD) of antifungal drugs were calculated according to published World Health Organization (WHO) guidelines and reflect the assumed average maintenance dose per day for a drug used for its main indication in adults [20].

*Candida* bloodstream infections and disseminated candidiasis were defined according to the European Organisation for Research and Treatment in Cancer (EORTC) criteria [21],[22]. Specifically, invasive candidiasis was defined as the isolation of a *Candida* species from a sterile specimen. All *Candida* isolates were identified in the hospital’s microbiology laboratory. Only the first episode in a patient with a mono-microbial infection was counted. Positive cultures of *Candida* species from urine, from rectal and oral swabs, or from respiratory secretions were always interpreted as colonisation. There is no systematic surveillance for *Candida* colonisation as part of ONKO-KISS; therefore, any recorded results from non-sterile specimens were not included in this analysis.

A panfungal polymerase chain reaction (PCR) assay that was developed in the hospital’s Clinical Microbiology Laboratory was used to verify the presence of clinically suspected candidiasis in a biopsy specimen when a blood culture remained negative. This PCR-assay uses ITS5 and ITS4 primers for amplification of ITS region 1 and region 2, followed by sequencing for species identification [23].

Prior to 2007, blood cultures were processed by using the automated BacT/ALERT® system. Since 2007, the BacT/ALERT 3D® Microbial Detection System (bioMérieux, Durham, North Carolina, USA) was used and the FA aerobic and FN anaerobic bottles processed according to the manufacturer’s instructions. *Candida* strains were identified by using standard microbiology techniques, including the use of the ID 32 C identification system (bioMérieux, Marcy-l’Étoile, France). From January 2003 through December 2004, MICs of antifungal drugs with respect to strains of *Candida* in samples were determined in an external microbiology laboratory that was affiliated with the University. Thereafter, the antifungal drug MICs were determined in the hospital’s Clinical Microbiology Laboratory by using the Sensititre YeastOne® colorimetric antifungal panel (TREK Diagnostic Systems Inc., Cleveland, Ohio, USA) and interpreted according to the 2008 breakpoints defined by the Clinical and Laboratory Standards Institute (CLSI), formerly known as the National Committee for Clinical Laboratory Standards (NCCLS).

Fisher’s exact test was used to compare the fluconazole susceptibility defined as MIC < or equal to 8 mg/L for all invasive *Candida* strains. We also compared the MICs of invasive *C. albicans* and *C. glabrata* strains respectively. Estimating a trend for *C. krusei* seemed not appropriate to us as three of the four strains with the same MIC occurred within the same year. Linear regression was applied to compare the incidence of different *Candida* isolates. Two-tailed p-values <0.05 were considered significant. Analyses were performed by using SPSS Statistical Software Version 21.0. The number of positive cultures in this study was too small a sample to perform additional meaningful statistical analyses.

## Results

### Patient characteristics

We had a total of 899 patients who were at least once hospitalized in the RIHU from January 2003 until December 2012. These patients spent a mean of 3977 bed days (BD) per year in the RIHU with a median duration of 26 days in the RIHU per patient (IQR 18–33). From July 2003 through December 2012, 753 of the 899 patients became registered in the ONKO-KISS as being neutropenic and underwent HSCT or, from 2008 onwards, were included because of their induction or consolidation chemotherapy. Most of them suffered from acute myeloid leukaemia. 612 were admitted for allogeneic or autologous stem cell transplantations and 134 were admitted for high-dose chemotherapies. Chemotherapy regimens included induction, re-induction, and consolidation chemotherapy (See Table 1 for patient characteristics of the RIHU ONKO-KISS population).

**Table 1:** **Baseline characteristics of patients hospitalized on RIHU and registered in ONKO-KISS from July 2003 through December 2012**

**Total number of patients**	753
Males	458 (60.8%)
Females	295 (39.2%)
**Median age in years**	
Males	49 (38–61 IQR)
Females	50 (38–59 IQR)
**Underlying conditions:**	
Acute myeloid leukaemia	304 (40.4%)
Acute lymphatic leukaemia	97 (12.9%)
Myelodysplastic syndrome	47 (6.2%)
Non-Hodgkin lymphoma	70 (9.3%)
Plasma cell disorders	83 (11%)
Underlying condition not in records	1 (0.1%)
**Treatment regimens:**	
Number of allogeneic HSCT recipients:	440
PBSC recipients	412 (93.6%)
BMT recipients	25 (5.7%)
Cord blood recipients	3 (0.7%)
Donor related/unrelated	242/198 (55.0%/45.0%)
Number of autologous HSCT recipients:	172
Number of patients undergoing induction, re-induction or conditioning chemotherapy:	123
Other chemotherapies (consolidation chemotherapy, or treatment not specified in database)	16

### Incidence and susceptibility of *Candida*species

There were a total of 28 invasive candidiasis episodes within 28 patients during this decade. The average age of these patients was 47 years. The majority (16/28; 57%) of these patients suffered from acute myeloid leukaemia (AML), and most patients underwent allogeneic HSCT (Table 2). Among the 612 HSCT recipients on RIHU candidaemia occurred in 4.6% corresponding to 4.6 candidaemia episodes per 100 transplantations (Tables 1 and 2). Two patients were each infected during one hospitalisation period with two different strains of *Candida* species and were therefore excluded from analysis. *Candida* species were isolated from blood cultures (n = 26), a skin biopsy (n = 1), and a liver biopsy (n = 1).

**Table 2:** **Characteristics of the 28 patients with invasive candidiasis in a single-centre study over 10 years**

**Total number of invasive candidiasis episodes**	28
**Distribution of invasive** ***Candida*** **species**	**-**
*C. albicans*	4
*C. glabrata*	8
*C. krusei*	4
*C. norvegensis*	3
*C. inconspicua*	2
*C. dubliensis*	2
*C. parapsilosis*	2
*C. kefyr, C. guilliermondii, C. tropicalis (*each 1*)*	3
**Gender**	
Number of males	12 (42.9%)
Number of females	16 (57.1%)
**Median age in years**	47 (31–63 IQR)
**Underlying conditions:**	
Number of patients with:	
Acute myeloid leukaemia	16
Acute lymphatic leukaemia	3
Myelodysplastic syndrome	3
Non-Hodgkin lymphoma and plasmocytoma (2 each)	4
Aplasia of unknown origin, hereditary mitochondropathy (1 each)	2
**Haematological treatments**	
Number of allogeneic HSCT recipients	15
PBSC recipients	13
BMT recipient	1
Cord blood recipient	1
Related/unrelated donor	1/14 (6.7%/93.3%)
Conditioning chemotherapy before HSCT	2
Induction chemotherapy	8
Other chemotherapies (e.g. consolidation, not specified)	3
**Other risk factors for invasive candidiasis**	
A stay in the ICU within 7 days prior to onset of invasive candidiasis	4
Number of patients in neutropenia (<500/uL) at time of invasive candidiasis	19
Median duration (days) of neutropenia until diagnosis of invasive candidiasis	13 (7–24 IQR)
Number of patients with CMV disease before invasive candidiasis episode	2
Number of patients with onset of GvHD before invasive candidiasis	5
**Outcome**	
Death (all cause) within 30 days after diagnosis of invasive candidiasis	10

The analysis of the 28 invasive candidiasis strains revealed 8 *C. glabrata* (29%), 4 *C. albicans* (14%), and 4 *C. krusei* (14%) as the most common strains (Table 2). Other species that were less often found included *C. norvegensis* (11%), *C. inconspicua, C. dubliensis*, and *C. parapsilosis* (7% each), *C. kefyr*, and *C. guilliermondii*, and *C. tropicalis* (4% each).

Only one haematology patient in this study suffered from hepato-splenic candidiasis. The culture from the patient’s liver biopsy remained negative, but the panfungal PCR assay was positive for *C. parapsilosis*.

**Figure 1:**
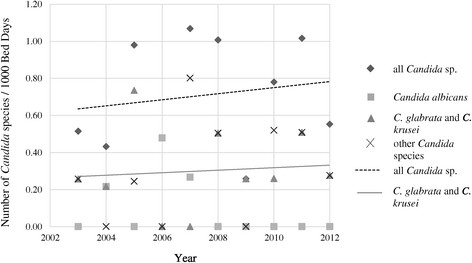
**Incidence of invasive Candidiasis on RIHU from 2003–2012.** Other *Candida* species: *C. norvegensis, C. inconspicua, C. dubliensis; C. parapsilosis, C. kefyr, C. guilliermondii, C. tropicalis.*

Overall, the median incidence for invasive candidiasis was 0.67 per 1000 bed days (IQR 0.47-1.01 per 1000 bed days) and we were unable to demonstrate a significant increase over the 10 years (Figure 1).

With linear regression analysis we found no significant increment in non-*albicans Candida* species versus *C. albicans* alone (p = 0.466). Similarly, when *C. glabrata* and *C. krusei* were taken together versus all other *Candida* species, the linear regression showed only a mild increase (p = 0.603), despite the fact that *C. glabrata* and *C. krusei* are usually considered as not susceptible to or intrinsically resistant to fluconazole. However, we found a trend towards more *C. glabrata* versus all other *Candida* species over this decade, but this was not statistically significant (p =0.124).

In 27 of the 28 isolates it was possible to test for fluconazole susceptibility. The *C. parapsilosis* isolate identified by PCR assay could not be tested because it did not grow in culture and there is currently no standard molecular susceptibility method that would apply to this strain.

**Figure 2:**
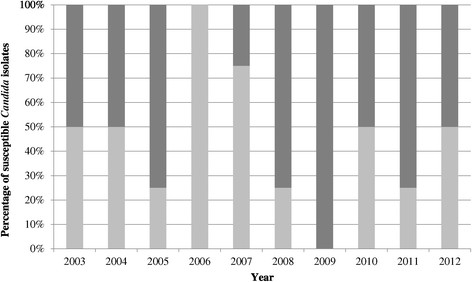
Susceptibility* of invasive ***Candida*** **isolates to fluconazole from 2003–2012.** Light grey bars = percentage of susceptible *Candida* species. ^*^Susceptibility is defined as a minimum inhibitory concentration (MIC) of < or equal to 8 mg/L (CLSI definition, 2008). CLSI = Clinical and Laboratory Standards Institute, formerly known as the National Committee for Clinical Laboratory Standards (NCCLS).

No trend toward reduced fluconazole susceptibility among invasive *Candida* isolates was found over the 10 years (Figure 2), with susceptibility being defined according to 2008 CLSI breakpoint definitions that consider isolates with a MIC of ≥8 mg/L to be non-susceptible (p = 0.162, 95% CI 0.8651- 3.67). The median MIC of the most common invasive strains was as follows (IQR): 0.19 mg/L (0.12 – 0.16 mg/L) for *C. albicans*, 12 mg/L (8 – 208 mg/L) for *C. glabrata* and 128 mg/L (44–128 mg/L) for *C. krusei*. A trend analysis did not show a significant increase in MIC. However, the number of data points precludes a meaningful interpretation of this analysis.

### Antifungal consumption

Regarding administration of antifungals at the RIHU we found that amphotericin B use decreased markedly from 2005 to 2008 (from 50 DDD/100 PD to 3.1 DDD/100 PD) coinciding with an increase of caspofungin use which has also a certain anti-mould activity but is better tolerated and less toxic than the deoxycholate form of amphotericine B. A re-increase in amphotericine use to a maximum of 38 DDD/100 PD in 2010 was likely due to the introduction of the new liposomal amphotericin B.

**Figure 3:**
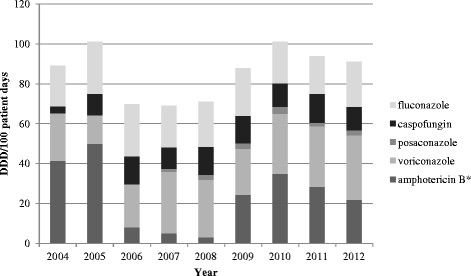
**Antifungal use on RIHU from 2004–2012 in defined daily dosages (DDD) per 100 patient days.** * Liposomal and conventional (cholesteryl sulfate complex) amphotericin B.

The use of voriconazole over the same time span exceeded the median amphotericin use slightly with 28.8 DDD/100 PD. Caspofungin consumption increased markedly from 3.5 DDD/100 PD in 2004 to 14.1 DDD/100 PD in 2006, but its use changed only minimally thereafter. The level of fluconazole use remained stable (median 22.6 DDD/100 PD) over the course of the study and reflects prophylactic use. Posaconazole was introduced in 2007 as an antifungal therapeutic agent, but it has rarely been applied as prophylaxis at this hospital. The consumption of antifungals is shown in Figure 3.

## Discussion

In the most recent European guidelines of the European Conference on Infections in Leukaemia (ECIL), administration of 400 mg fluconazole once daily is graded as an AI recommendation for HSCT recipients in the initial neutropenic phase if a mould-directed diagnostic approach is provided and is graded as a CI recommendation for patients with chronic GvHD and patients with acute leukaemia during induction chemotherapy [24]. With intention to reducing morbidity and mortality due to *Candida* diseases in HSCT recipients until 100 days post HSCT fluconazole 400 mg once daily has also been ascribed an AI recommendation by the European Society of Clinical Microbiology and Infectious Diseases (ESCMID) [25]. In the American guidelines 400 mg of fluconazole once daily has an AI recommendation grade for all patients with haematological malignancies or who are recipients of HSCT [26],[27]. In several retrospective studies, the incidence of invasive candidiasis in patients who were undergoing HSCT varied within a range of 0.9% to 4.6% depending on the country, the centre where the studies took place, and the percentage of allograft recipients in the studies, even though the majority of the studies used 400 mg of fluconazole on a daily basis as antifungal prophylaxis [1],[28]. In this ten-year study, however, we found a remarkably stable median incidence of invasive candidiasis with 0.67 per 1000 BD and an incidence of 4.6% among all HSCT recipients using only 400 mg fluconazole once a week as antifungal prophylaxis. This finding was unexpected. Albeit there could be several other factors playing a role in preventing invasive candidiasis like the strict reverse isolation practice, optimized central venous catheter care, or high level of hand hygiene compliance, but these components did not change considerably over this time period and, therefore, seem to play a minor role. We postulate that this weekly dosage of fluconazole indeed reduces mucocutaneous colonisation with *Candida* species during profound neutropenia and, therefore, preventing sufficiently invasive candidiasis in a setting with general low prevalence of invasive fungal infections.

The distribution of *Candida* species in this analysis did not vary significantly over the study period, but it differs from data in other published epidemiological studies in Europe that involved a 400 mg dose of fluconazole on a daily basis. Surveillance data from Europe listed the four most common *Candida* species as *C. albicans* (34.6%), *C. glabrata* (9.7%), *C. parapsilosis* (14.8%), and *C. tropicalis* (17.9%) in a total of 257 haematology patients [29]. The results of this study also differ from other studies that used lower daily dosages of fluconazole. In Safdar et al. [6], who used a mean fluconazole dose of 150 mg/day (100–200 mg/day), the distribution in 15 episodes of candidaemia was *C. glabrata* (53.3%), *C. krusei* (33.3%), *C. parapsilosis* (13.3%), and no *C. albicans*. This study’s distribution in 28 episodes of invasive candidiasis is *C. glabrata* (29%), *C. albicans* (14%), and *C. krusei* (14%).

The mild trend toward a higher incidence of *C. glabrata* which is known to be dose dependent fluconazole susceptible as well as a decreased incidence of invasive *C. albicans* in the most recent years is consistent with other studies, even when their regimens were once-daily use of fluconazole as a prophylaxis [17],[30]. However, the absence of *C. krusei* in the last years of the whole decade is rather unexpected. Admittedly with a small sample set, it was impossible to demonstrate a significant shift towards less fluconazole susceptible species.

This study and the previous study by our colleagues [16] have shown that this unique regimen of antifungal prophylaxis (administration of fluconazole on a weekly basis) in combination with an early diagnostic-driven approach to treatment for suspected mould infections is safe and also that it might contribute to a relatively stable incidence of invasive candidiasis that is consistent with the incidence over time reported in other studies.

Although it is a single-centre study, it seems to have a representative cohort of patients that is similar to cohorts in other studies reported in the literature with patients registered only when neutropenic and undergoing induction chemotherapy or HSCT.

The major limitations were the small sample size of patients with invasive candidiasis precluding us from providing additional meaningful statistics and that we lack data on empirical antifungal treatment over the study period. In addition, there is neither an active nor a historical control group without prophylaxis with which we could compare this sample population because of the strong advice for antifungal prophylaxis. Own former data about incidence of invasive candidiasis before introducing this regimen are lacking. There were centres in Switzerland without providing prophylaxis to their haemato-oncology patients and centres with different prophylaxis such as low dosages of liposomal amphotericin B i.v.; however, the heterogeneity of this population in important predictors, e.g. absence of allogeneic HSCT, and that the centres abandoned their strategy or switched to a different antifungal prophylaxis renders a comparison of the incidence and distribution of invasive *Candida* species with our data difficult. Monitoring of trough levels of fluconazole was not routinely performed in our hospital.

The strengths of this study therefore are (1) a 10-year study period that comprises 753 patients at high risk for invasive candidiasis due to the treatment regimens for their underlying conditions, and (2) the use of standardized criteria for diagnosing invasive candidiasis, with 97% of the samples verified by a positive culture from a sterile site.

## Conclusions

This unique regimen of administering fluconazole weekly showed neither a significant increase in the incidence of invasive candidiasis, or an emergence of fluconazole resistance, nor a significant shift toward non-*albicans Candida* species. In upcoming guidelines, the current recommendation for fluconazole prophylaxis in HSCT patients may be revised due to strong evidence for the use of posaconazole and voriconazole as effective prophylactic agents in this population. But a once weekly dose of fluconazole may still be a safe and effective alternative prophylaxis that can potentially reduce exposure to antifungal drugs and lower drug toxicities and drug interactions in these high-risk patients provided that the general prevalence of invasive fungal infections in this setting is low.

### Ethical considerations

The study has been conducted as part of the continuous quality improvement program and was exempt from ethical approval (Ethikkommission Nordwest- und Zentralschweiz (EKNZ), formerly known as Ethikkommission beider Basel, Basel, Switzerland).

## Authors’ contributions

DV drafted the manuscript, and was substantially involved in acquisition, analysis and interpretation of data. MW, CO, RF, DH and JRP have been involved in critically revising the manuscript for important intellectual content. AFW conceived of the study and participated in its design and helped to draft the manuscript. All authors read and approved the final version of the manuscript.

## Authors’ original submitted files for images

Below are the links to the authors’ original submitted files for images. Authors’ original file for figure 1 Authors’ original file for figure 2 Authors’ original file for figure 3
