# Supplementary figures and images for: Weekly use of fluconazole as prophylaxis in haematological patients at risk for invasive candidiasis
Source: BMC Infect Dis. 2014 Nov 11;14:573. doi: 10.1186/s12879-014-0573-5 (PMC4233028; doi:10.1186/s12879-014-0573-5)

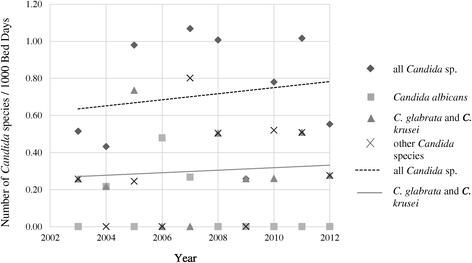

Supplement: Supplementary file 1 — Authors’ original file for figure 1 [file 12879_2014_573_MOESM1_ESM.gif]

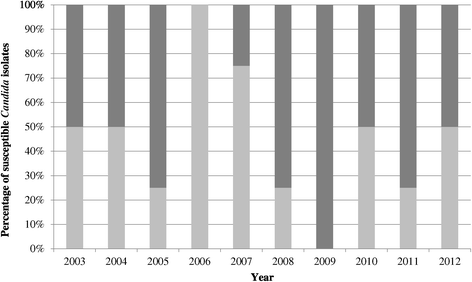

Supplement: Supplementary file 2 — Authors’ original file for figure 2 [file 12879_2014_573_MOESM2_ESM.gif]

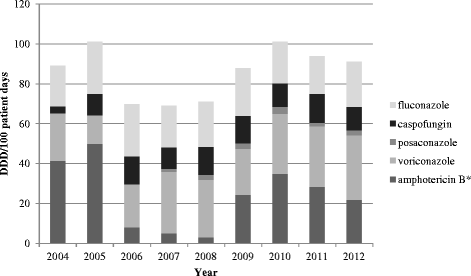

Supplement: Supplementary file 3 — Authors’ original file for figure 3 [file 12879_2014_573_MOESM3_ESM.gif]
